# The impact of pharmacist-led antimicrobial stewardship program on antibiotic use in a county-level tertiary general hospital in China: A retrospective study using difference-in-differences design

**DOI:** 10.3389/fpubh.2022.1012690

**Published:** 2022-10-03

**Authors:** Ying Wang, Chongchong Zhou, Chengying Liu, Shuanghai Liu, Xiaoliang Liu, Xin Li

**Affiliations:** ^1^Department of Infection Management, The First Affiliated Hospital of Soochow University, Suzhou, China; ^2^School of Health Policy and Management, Nanjing Medical University, Nanjing, China; ^3^Department of Clinical Pharmacy, School of Pharmacy, Nanjing Medical University, Nanjing, China; ^4^Department of Respiratory Medicine, The Affiliated Jiangyin Hospital of Nantong University, Jiangyin, China; ^5^Department of Hepatobiliary Surgery, The Affiliated Jiangyin Hospital of Nantong University, Jiangyin, China; ^6^Department of Infection Management, The Affiliated Jiangyin Hospital of Nantong University, Jiangyin, China; ^7^Center for Global Health, School of Public Health, Nanjing Medical University, Nanjing, China

**Keywords:** antibiotics use, antimicrobial stewardship, pharmacist, difference-in-differences, county-level general hospital

## Abstract

**Background:**

Inappropriate use of antibiotics has become a major driver for the spread of antimicrobial resistance globally, particularly common in China. Antimicrobial stewardship programs are effective in optimizing antimicrobial use and decreasing the emergence of multi-drug-resistant organisms, and the pharmacist has performed a leading role in this program.

**Objective:**

To evaluate the impact of antimicrobial stewardship programs driven by pharmacists on antibiotic consumption and costs and the appropriateness of antibiotic use.

**Methods:**

A single-center retrospective quasi-experimental design was conducted in two independent hepatobiliary surgery wards and two independent respiratory wards in a county-level tertiary general hospital in Jiangsu, China. Each intervention group was served with antimicrobial stewardship programs with prescriptions audit and feedback, antibiotics restriction, education, and training. The propensity score matching method was employed to balance confounding variables between the intervention group and control group, and a difference-in-differences analysis was used to evaluate the impact of antimicrobial stewardship programs. The primary outcome was measured by scores of rationality evaluation of antibiotics.

**Results:**

The DID results demonstrated that the implementation of the antimicrobial stewardship programs was associated with a reduction in the average length of hospital stay (coefficient = −3.234, *p* = 0.006), DDDs per patient (coefficient = −2.352, *p* = 0.047), and hospitalization costs (coefficient = −7745.818, *p* = 0.005) in the hepatobiliary surgery ward, while it was associated with a decrease in DDDs per patient (coefficient = −3.948, *p* = 0.029), defined daily doses per patient day (coefficient = −0.215, *p* = 0.048), and antibiotic costs (coefficient = −935.087, *p* = 0.014) in the respiratory ward. The program was also associated with a decrease in rationality evaluation scores (*p* < 0.001) in two wards.

**Conclusion:**

The result reveals that the implementation of the antimicrobial stewardship programs is effective in reducing the length of hospital stay, decreasing antibiotics consumption and costs, and improving the appropriateness of antimicrobial use such as decreasing irrational use of cephalosporins, reducing combinations, and improving timely conversion. However, great attention ought to be paid to the improper use of broad-spectrum antibiotics. The government is responsible for providing sustainable formal education for pharmacists, and more funding and staff support to promote antimicrobial stewardship programs.

## Introduction

Overuse of antibiotics has become a major contributor to the emergence and rapid spread of antimicrobial resistance (AMR) (1). As multi-drug resistance has emerged in plenty of species, few antibiotics in the development pipeline make AMR a growing public health crisis on a global level. Unfortunately, it is estimated that 20–50% of prescriptions for antibiotics were inappropriate or unnecessary, and if no action was taken, by 2050 AMR could cause as many as 10 million deaths per year and the economic burden of deaths could reach 100 trillion dollars per year (2, 3).

For several decades, China has experienced an extensive threat of AMR due to antibiotic abuse and was the second largest antimicrobial consumer worldwide (4). In 2011, the National Health and Family Planning Commission (NHFPC) of China put forward the “National Special Stewardship in the Clinical Use of Antibiotics” to improve the use of antibiotics. The campaign made some progress and had positive effects on decreasing the consumption and intensity of antibiotic use (5); however, due to the strong administrative intervention and little attention to attitude and practice on prescriptions of physicians, the trends of AMR had not decreased as expected (5). For instance, the percentage of Escherichia coli resistant to third-generation cephalosporins and quinolones was 59 and 54, respectively, in 2015 (6), which suggested that effective antimicrobial management was urgently needed in China.

Antimicrobial stewardship (AMS) was the foundation of a hospital's antimicrobial stewardship programs (ASPs) and has been successfully implemented in most hospitals in developed countries. The primary goal of the ASPs is to optimize antimicrobial use and patient outcomes while decreasing the spread of multi-resistant infections and reducing adverse effects and some other unintended consequences of antibiotics (7). As an integral part of the antimicrobial stewardship team, pharmacists being active members of the healthcare system have a significant role in implementing strategies and monitoring performance to achieve the primary goals of ASPs. In specific, pharmacists were responsible for developing evidence-based guidelines, reviewing prescriptions and providing feedback, and monitoring antibiotic use and are capable of delivering education and training (8, 9).

Due to the positive effects on abuse and rational use of antibiotics, the Chinese government has attached importance to pharmacist-led antimicrobial stewardship. Only a few studies evaluated the effects of pharmacist-led antimicrobial stewardship in China. A multi-center prospective cohort study showed that pharmacist-driven antimicrobial stewardship had a lower mortality rate and more optimized antimicrobial use in intensive care units (ICUs) (10). However, only the patients in ICUs and pediatric patients were included in the above-mentioned studies. Moreover, the previous studies focused on the effects of ASP in urban hospitals. However, in China, urban–rural disparities exist in antibiotics use and the healthcare services system. Nevertheless, the effects of pharmacist-led antimicrobial stewardship established in county-level tertiary general hospitals in rural areas have not been estimated. Therefore, this study aimed to evaluate the impact of pharmacist-led antimicrobial stewardship on antibiotic consumption and costs and the appropriateness of antibiotic use in internal medicine and surgery departments of a county-level general hospital in Jiangsu, China.

## Methods

### Setting

A single-center quasi-experimental study was conducted in Jiangyin, Jiangsu, China, a 2200-bed, teaching, general hospital. The hospital ranked third among the top 100 county hospitals in China in 2020, and it provides comprehensive medical treatment, prevention, healthcare, and rehabilitation care.

### Study design

The study period was over 20 months, with a pre-intervention phase from March 2018 to October 2018, an ASP implementation phase in January 2019, and an intervention evaluation phase from March 2019 to October 2019. It was conducted in two hepatobiliary surgery wards: one was regarded as an intervention group and the other was the control group, and two respiratory wards: one was an intervention group and the other was the control group. Each intervention group was served with ASPs by an antimicrobial management group, while each control group received standard strategies performed by physicians without pharmacist involvement.

Patients admitted to the hospital with antibiotic treatment were included in the study period. Among them, those who were diagnosed with cholecystectomy and choledocholithotomy in the hepatobiliary surgery ward and with chronic obstructive pulmonary disease (COPD), bronchial asthma, pneumonia, bronchitis, and respiratory failure in the respiratory ward were eligible for the study.

### Ethical consideration

This study was approved by the Ethical Committee of Nanjing Medical University (Grant number: 2020103) and conducted following the Declaration of Helsinki guidelines.

### ASPs intervention

In January 2019, an ASP was introduced in the respiratory ward and hepatobiliary surgery ward of a county-level tertiary general hospital, aiming to improve appropriateness in antibiotic use and reduce antibiotic consumption. The antimicrobial management team was composed of a full-time infectious diseases-trained clinical pharmacist as its core, also with physicians, clinicians, infectious disease specialists, microbiologists, and administrators, and included a leadership group responsible for work deployment and supervision. Generally, an infectious disease physician-led or administration department of nosocomial infection-led antimicrobial stewardship was the main model in hospitals in China, whereas pharmacists played a leading role in AMS in our study for the following reasons: (1) the pharmacist participated in ward rounds per day so that they could communicate with physicians closely, (2) the pharmacists had sufficient knowledge to monitor the drug use of patients and give individual dosing schedules, and (3) the pharmacists were responsible for the education and training of physicians, microbiologists, administrators, and patients. The ASPs were designed by a pharmacist-led antimicrobial team based on the updated Chinese Guidelines for the Clinical Application of Antimicrobial Agents, and multifaceted interventions included:

#### Audit and feedback

An audit of antibiotic prescriptions with timely feedback to prescribers was a core strategy in antimicrobial stewardship, which included: (a) a daily review of prescriptions with direct feedback outlining suggestions on potential inappropriate use of antibiotics, rationality for the recommendation, and information on antibiotics-related adverse reaction to prescribers; (b) daily ward rounds with physicians to assess the patients' diagnosis, medication, and microbiological results, and giving advice to the physician to determine the optimal drug treatment; and (c) a discussion on possible recommendations for complex cases with an infectious disease specialist, physicians, and microbiologists.

#### Formulary restriction

A defined hospital formulary was established based on the antibiotic classification management policy which classified antibiotics into first-line, second-line, and third-line antibiotics authorization. The interventions on formulary restriction included: (a) limiting the use of third-line antibiotics unless there was a clear indication; (b) giving prescriptive authority to physicians in different positions to prescribe different classes of antibiotics; and (c) incorporating restrictions into computer physician order entry to trigger initial review when the inappropriate rationale for antibiotic selection or physicians without authority for particular antibiotics occurred.

#### Education and training

Pharmacists played a significant role in delivering education and training on antimicrobial stewardship to professionals, patients, and members of the public (9, 11). In the proceeding of ASPs implementation, the pharmacists (a) organized seasonal educational meetings with the data sharing of the change in antibiotic resistance, antibiotic consumption, and costs and (b) organized education and training of medical staff on the rational use of antibiotics.

#### Reward and punishment

Reward and punishment assessment of the ASPs on antibiotic consumption and costs and appropriate antimicrobial use was performed by pharmacists based on the reported data.

### Data collection and outcomes

Data were obtained from the hospital information system (HIS) for each inpatient on (1) demographic characteristics: age and gender; (2) clinical information: diagnosis, transition, complications, bacterial culture or not, drug sensitivity test or not, etiology submission or not, procalcitonin value, C-reactive protein value, admission date, and discharge date; (3) antibiotic use: substance name, unit strength, pack size, defined daily dose (DDD), the route of administration, and the date dispensed; and (4) costs: antibiotic costs and hospitalization costs. The HIS was linked with an inpatient database, pharmacy database, and microbiology database, and the data were measured repeatedly by two authors during the study period to assure the data quality and adequacy.

Our primary outcome was measured by scores of rationality evaluation of antibiotics. After hospital discharge, a part of medical records for inpatients in the two groups selected through equidistant sampling was reviewed by two infection specialists, and the phenomena of inappropriate antibiotic use were evaluated based on a scoring system involving items (Table 1). The item was assigned one point if an inappropriate antibiotic use was identified and then multiplied by the corresponding weight calculated through an analytic hierarchy process to obtain final scores.

**Table 1:** Scoring system involving weights of different items.

**Items**	**Therapeutic use of antibiotics**	**Perioperative antimicrobial prophylaxis**
Indication	0.37	0.41
Choice	0.23	0.25
Dosage	0.12	0.11
Dosing schedule	0.10	0.16
Duration	0.07	0.17
Conversion	0.06	NA
Combination	0.05	NA

NA, not available.

Secondary outcomes were antibiotic consumption, antibiotic costs, and medical service efficiency. As our study concentrated on the effect of ASP intervention on the appropriate use of antibiotics during the hospitalization period, the discharge medication was not taken into consideration in the study. Antibiotic consumption was measured by DDDs per patient and DDDs per patient per day.


$$\begin{array}{l} \text{ DDDs}=\;\frac{\text{unit strength}\times \text{pack size}}{\text{DDD}}\\ \;\times \text{ unmber of package of antibiotics},\\ \text{DDDs per patient}=\;\frac{\text{DDDs}}{\text{number of inpatient hospitalizations}},\\ \text{ DDDs per}\\ \text{ patient per day}=\;\frac{\text{DDDs}}{\begin{array}{c} \text{number of inpatient hospitalizations}\\ \times \text{ average LOS}\; \end{array}}. \end{array}$$

Costs were measured by antibiotic costs, daily costs, and hospitalization costs. Antibiotic costs were computed by the expense for all antibiotics of patients during the study period, while hospitalization costs included costs of all drugs (antibiotics included), examination expenses, surgery expenses, registration fees, etc., and the daily costs were computed by antibiotics costs divided by corresponding DDDs.


$$\begin{array}{l} \text{Daily costs}=\frac{\text{total antibiotic costs}}{\text{DDDs}}. \end{array}$$

Medical service efficiency was measured by the average length of hospital stay (LOS), LOS was calculated by subtracting the admission date from the discharge date, and the average LOS was calculated by the length of stay divided by the number of inpatient hospitalizations.


$$\begin{array}{l} \text{Average LOS}=\frac{\text{total LOS}}{\text{number of inpatient hospitalizations}}. \end{array}$$

### Statistical analysis

Propensity score matching (PSM), as a statistical method for dealing with sample selectivity bias, was first proposed by Rosenbaum and Rubin in 1983 (12). The propensity score is defined as the probability of being eligible for ASPs, and a logistic regression was used to estimate propensity scores as a function of a set of covariates that could influence the impact on antibiotic consumption and costs and the likelihood of improving antibiotic use. In this study, the covariates including age, gender, transition, and diagnosis were incorporated into regression and nearest-neighbor matching with one-to-one matches could identify individuals in the control group with similar characteristics as those served with ASPs based on propensity scores. The PSM allowed the selection of the appropriate control group, which helped to solve possible endogenous problems and demonstrate the robustness of the results.

It is widely acknowledged that a difference-in-differences (DID) method is a quasi-experimental technique for policy evaluation. We perform a DID analysis to study the differential impacts of ASPs intervention and construct the following regression model:


$$\begin{array}{l} Y_{it}=\beta_{0}+\beta_{1}\times time_{t}+\beta_{2}\times group_{i}+\beta_{3}\times time_{t}\times group_{i}\\ \;+\;\gamma \times X_{it}+\varepsilon_{it} \end{array}$$

where *Y*_*it*_ stands for the rationality evaluation, consumption, and costs of antibiotics; the key coefficient β_3_ is calculated by a DID model and reflects the effect of ASPs intervention; the variable *group*_*i*_represents the group dummy variable and *group*_*i*_=1 indicates the treatment group, whereas *group*_*i*_=0 indicates the control group, *t* indicates the month; *time*_*t*_is the time dummy variable where pre-intervention (March 2018–October 2018) is 0, whereas post-intervention (March 2019–October 2019) is 1; the variable *time*_*t*_×*group*_*i*_ denotes the interaction between time and groups; *X*_*it*_ means a series of covariates of resident *i* at time *t*; and ε_*it*_is the residual error.

To estimate the effects of ASPs on the rationality evaluation, consumption, and costs of antibiotics in China, we employed a PSM-DID model rather than a matching method or a DID analysis alone. The combination of two methods can be applied to dealing with relatively weak assumptions to help DID meet common trends on observables (13).

Statistical analyses were conducted using Stata, version 14.0 (Stata Corp., College Station, TX, USA), and *P* < 0.05 was considered statistically significant. All expenses in this study were measured by CNY.

## Results

### Study population

During the 10-month intervention, Table 2 illustrates that in the hepatobiliary surgery ward, there were 207 and 839 hospitalizations identified in the intervention and control groups, respectively. Most patients were female (564, 53.92%), and there existed a difference in gender between the two groups (*p* = 0.011). Besides, the transition of patients (*p* < 0.001) and the diagnosis (*p* < 0.001) were different between the intervention and control groups. After propensity matching, the sample size decreased to 392 and each group had 196 patients for the study, and the propensity was effective in reducing differences for all covariates.

**Table 2:** Baseline characteristics of patients in the hepatobiliary surgery ward and respiratory ward.

	**Before matching**			**After matching**		
**Hepatobiliary surgery ward**	**Control group** ** (*n* = 839)**	**Intervention group** ** (*n* = 207)**	***P-*value**	**Control group** ** (*n* = 196)**	**Intervention group** ** (*n* = 196)**	***P-*value**
Male, *n* (%)	387 (46.13)	95 (45.89)	0.011	105 (53.57)	91 (46.43)	0.157
Age, mean ± SD	54.52 ± 14.66	54.88 ± 15.05	0.752	58.79 ± 13.96	58.37 ± 13.81	0.311
Transition, better, *n* (%)	811(96.66)	191 (92.27)	< 0.001	185 (94.39)	186 (94.90)	0.823
**Diagnosis**, ***n*** **(%)**						
Cholecystectomy, *n* (%)	694 (82.72)	168 (81.16)	< 0.001	170 (86.73)	165 (84.18)	0.474
Choledocholithotomy, *n* (%)	145 (17.28)	39 (18.84)		26 (13.27)	31 (15.82)	
**Respiratory ward**	**Control group** **(*****n*** = **1516)**	**Intervention group** **(*****n*** = **561)**	***P-*** **value**	**Control group** **(*****n*** = **559)**	**Intervention group** **(*****n*** = **559)**	***P-*** **value**
Male, *n* (%)	975 (64.31)	335 (60.43)	< 0.001	338 (60.47)	338 (60.47)	0.572
Age, mean ± SD	64.10 ± 16.59	62.59 ± 17.15	0.047	62.77 ± 17.05	62.59 ± 17.18	0.861
Transition, better, *n* (%)	1439 (94.92)	533 (95.01)	< 0.001	543 (97.14)	533 (95.35)	0.513
**Diagnosis**, ***n*** **(%)**						
COPD, *n* (%)	341 (22.49)	96 (17.11)	< 0.001	105 (18.78)	95 (16.99)	0.435
Bronchial Asthma, *n* (%)	91 (6.00)	9 (1.60)	< 0.001	17 (3.04)	9 (1.61)	0.112
Pneumonia, *n* (%)	513 (33.84)	244 (43.49)	< 0.001	231 (41.32)	244 (43.65)	0.432
Bronchitis, *n* (%)	195 (12.86)	41 (7.31)	< 0.001	40 (7.16)	41 (7.33)	0.908
Respiratory failure, *n* (%)	376 (24.80)	171 (30.48)	< 0.001	166 (29.70)	170 (30.41)	0.794

As shown in Table 2, in the respiratory ward there were 564 and 1523 hospitalizations enrolled in the intervention group and control group, respectively. The control group had relatively older patients (64.10 ± 16.59, *p* = 0.047), and there was a gender difference (*p* < 0.001), the transition of patients (*p* < 0.001), and diagnosis (*p* < 0.001) between the two groups. After propensity matching, the sample size decreased to 1118 and all covariates were well balanced between the two groups.

### Antibiotic consumption and costs and LOS before and after the ASP intervention

On average, 1244.55 CNY were spent on antibiotic costs per patient before and after the intervention throughout the study period in the hepatobiliary surgery ward: 964.81 CNY in the intervention group for 6.61DDDs per patient and 1336.33 CNY in the control group for 7.55 DDDs before the intervention and 875.11 CNY in the intervention group for 6.78 DDDs per patient and 1711.17 CNY in the control group for 10.38 DDDs after the intervention. As illustrated in Table 3, the DDDs per patient increased in the two groups and antibiotic costs decreased in the intervention group, but increased in the control group: a 2.57% increase in DDDs and a 9.30% decrease in antibiotic costs in the intervention group, but a 37.48% increase in DDDs and a 28.5% increase in antibiotic costs in the control group. In addition, the DDDs per patient day and daily costs decreased in the two groups: a decrease rate of 5% in DDDs per patient day and a 4.74% decrease in daily costs in the intervention group, but a 7.69% decrease in DDDs per patient day and a 5.96% decrease in daily costs in the control group. After the implementation of ASPs, the average LOS increased by 5.09% and 41.88% in the intervention and control groups, respectively.

**Table 3:** Mean of the consumption and costs of antibiotics and LOS in two wards.

**Outcomes**	**Total**	**Pre-intervention (2018.1-2018.10)**		**Post-intervention (2019.1-2019.10)**		**Change rate (%)**	
		**Intervention group(n_1_)**	**Control group(n_2_)**	**Intervention group(n_3_)**	**Control group(n_4_)**	**Intervention group**	**Control group**
**In the hepatobiliary surgery ward (n**_**1 =**_ **76, n**_**2 =**_ **69, n**_**3 =**_ **120, n**_**4 =**_ **127)**							
Average LOS	10.50	8.45	9.67	8.88	13.72	5.09	41.88
DDDs per patient	8.05	6.61	7.55	6.78	10.38	2.57	37.48
DDDs per patient day	0.76	0.80	0.78	0.76	0.72	−5.00	−7.69
Antibiotic costs	1244.55	964.81	1336.33	875.11	1711.17	−9.30	28.05
Daily costs	138.17	134.67	151.42	128.29	142.40	−4.74	−5.96
Hospitalization costs	21114.53	16752.25	17660.54	18740.56	27844.73	11.87	57.67
**In the respiratory ward (n**_**1 =**_ **176, n**_**2 =**_ **206, n**_**3 =**_ **383, n**_**4 =**_ **353)**							
Average LOS	8.83	9.67	7.82	9.15	8.67	−5.38	10.87
DDDs per patient	16.81	18.64	14.14	17.42	16.87	−6.55	19.31
DDDs per patient day	1.90	2.06	1.90	1.84	1.89	−10.68	−0.53
Antibiotic costs	2057.63	2620.67	1673.39	2037.22	2025.02	−22.26	21.01
Daily costs	118.98	143.03	117.55	115.18	111.52	−19.47	−5.13
Hospitalization costs	10502.22	10534.39	10592.98	10439.25	10406.77	−0.90	−1.76

LOS, length of hospital stay; DDDs, defined daily doses.

Averagely, 2057.63 CNY were spent on antibiotic costs per patient throughout the study period in the respiratory ward: 2620.67 CNY in the intervention group for 18.64 DDDs per patient and 1673.39 CNY in the control group for 14.14 DDDs in the pre-intervention period and 2037.22 CNY in the intervention group for 17.42 DDDs per patient and 2025.02 CNY in the control group for 16.87 DDDs in the post-intervention period. Meanwhile, the DDDs per patient and antibiotic costs decreased in the intervention group, but increased in the control group: a 6.55% decrease in DDDs and a 22.26% decrease in antibiotic costs in the intervention group, but a 19.31% increase in DDDs per patient and a 21.01% increase in antibiotic costs in the control group. In addition, the DDDs per patient day and daily costs decreased in the two groups: a decrease rate of 10.68% in DDDs per patient day and a 19.47% decrease in daily costs in the intervention group, but a 0.53% decrease in DDDs per patient day and a 5.13% decrease in daily costs in the control group. Compared with the pre-intervention period, the average LOS in the post-intervention group declined by 5.38% in the intervention group, but zoomed in the control group.

### Effects of ASP intervention on antibiotic consumption and costs and LOS

The results of the effects of ASPs intervention on antibiotic consumption and costs and LOS are illustrated in Table 4. The coefficient evaluation represents the interaction term between time and group variables, indicating the actual effect of the intervention. The results demonstrated that the ASPs intervention contributed to a decrease in LOS, DDDs per patient, and hospitalization costs in the hepatobiliary surgery ward and the coefficients were −3.234 (*p* = 0.006), −2.352 (*p* = 0.047), and −7745.818 (*p* = 0.005), respectively. In addition, the ASPs intervention also had a positive effect on DDDs per patient, DDDs per patient day, and antibiotic costs with coefficients of −3.948 (*p* = 0.029), −0.215 (*p* = 0.048), and −935.087 (*p* = 0.014), respectively, in the respiratory surgery ward. Although the ASPs intervention resulted in a decrease in DDDs per patient day and antibiotic costs in the hepatobiliary surgery ward and a decrease in LOS, daily costs, and hospitalization costs in the respiratory ward, there is no statistical significance in the above variables.

**Table 4:** DID results from liner regression analyses in two wards.

**Variable**	**Coefficient**	***p***	**RSE**	**95%CI**	
				**Lower bound**	**Upper bound**
**In the hepatobiliary surgery ward**
Average LOS	−3.234	**0.006**	6.290	−5.519	−0.949
DDDs per patient	−2.352	**0.047**	6.154	−4.676	−0.028
DDDs per patient day	−0.061	0.365	0.318	−0.194	0.071
Antibiotic costs	−404.675	0.197	1578.000	−1020.525	211.174
Daily costs	3.854	0.733	48.498	−18.395	26.068
Hospitalization costs	−7745.818	**0.005**	15690.000	−13078.780	−2412.860
**In the respiratory surgery ward**
Average LOS	−0.874	0.383	6.989	−2.841	1.093
DDDs per patient	−3.948	**0.029**	13.939	−7.489	−0.407
DDDs per patient day	−0.215	**0.048**	0.898	−0.429	−0.002
Antibiotic costs	−935.087	**0.014**	2887.200	−1679.427	−190.746
Daily costs	−21.821	0.214	106.840	−56.240	12.598
Hospitalization costs	−2458.432	0.130	10954.000	−5639.068	722.204

LOS, length of hospital stay; DDDs, defined daily doses; DID, difference-in-differences. Bold values, indicating *p* < 0.05.

### Estimation of scores of rationality evaluation of antibiotics

As shown in Table 5, concerning the great volume of medical records and limited time of reviewers, equidistant sampling with an interval of one patient was used and 196 medical records of patients (98 for the intervention group and 98 for the control group) were reviewed and evaluated by infection specialists in the hepatobiliary surgery ward. But, in the respiratory wards, equidistant sampling with an interval of five patients was employed and 186 medical records (93 for the intervention group and 93 for the control group) were reviewed. The results of the scores of rationality evaluation of antibiotics and the estimation of the ASPs intervention are shown in Table 5. On average, in the hepatobiliary surgery ward the rationality evaluation scores decreased by 0.145 in the intervention group, but increased by 0.007 in the control group after the implementation of ASPs. In the respiratory ward, the average rationality evaluation score was 0.316 and decreased to 0.033 in the intervention group after the ASPs, while the scores decreased from 0.209 to 0.196 in the control group. Moreover, the results showed that the ASPs intervention had a positive effect on the rationality evaluation scores in two wards (both *p* < 0.001), and the declining scores in the respiratory ward were more than those in the hepatobiliary surgery ward.

**Table 5:** Mean of the scores of rationality evaluation of antibiotics and DID results in two wards.

	**Hepatobiliary surgery ward**				**Respiratory ward**			
**Scores**	**pre-intervention**	**post-intervention**	**diff**	***P***	**pre-intervention**	**post-intervention**	**diff**	***P***
Intervention group	0.195	0.050	−0.145	< 0.001	0.316	0.033	−0.283	< 0.001
Control group	0.211	0.218	0.007		0.209	0.196	−0.013	
Regression–based DID			−0.152	< 0.001			−0.270	< 0.001
RSE			0.148				0.205	
Adj-*R*^2^			0.192				0.186	

### Analysis of inappropriate use of antibiotics based on rationality evaluation

**Figure 1:**
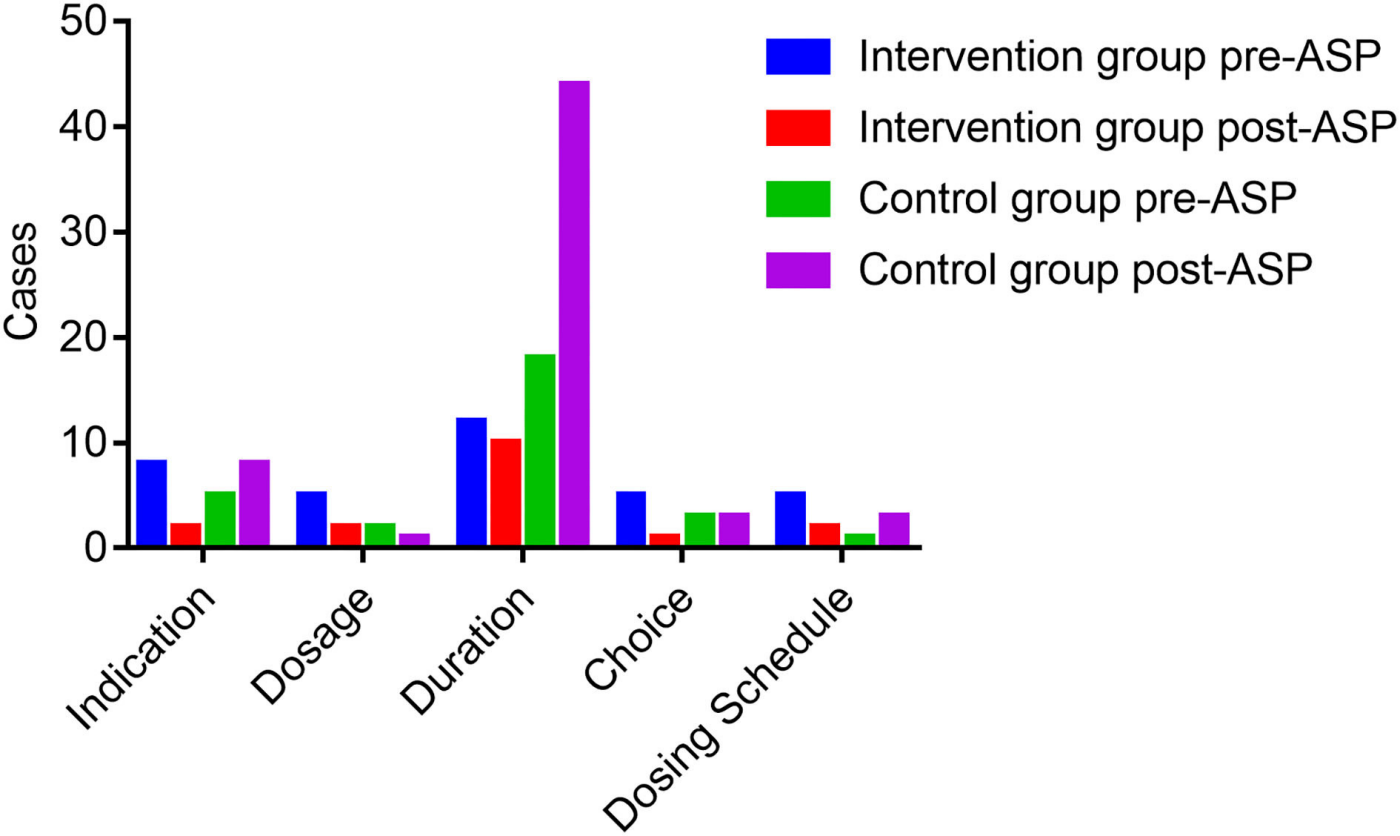
Inappropriate use of antibiotics before and after the ASPs implementation in the hepatobiliary surgery ward. ASPs, antimicrobial stewardship programs.

**Figure 2:**
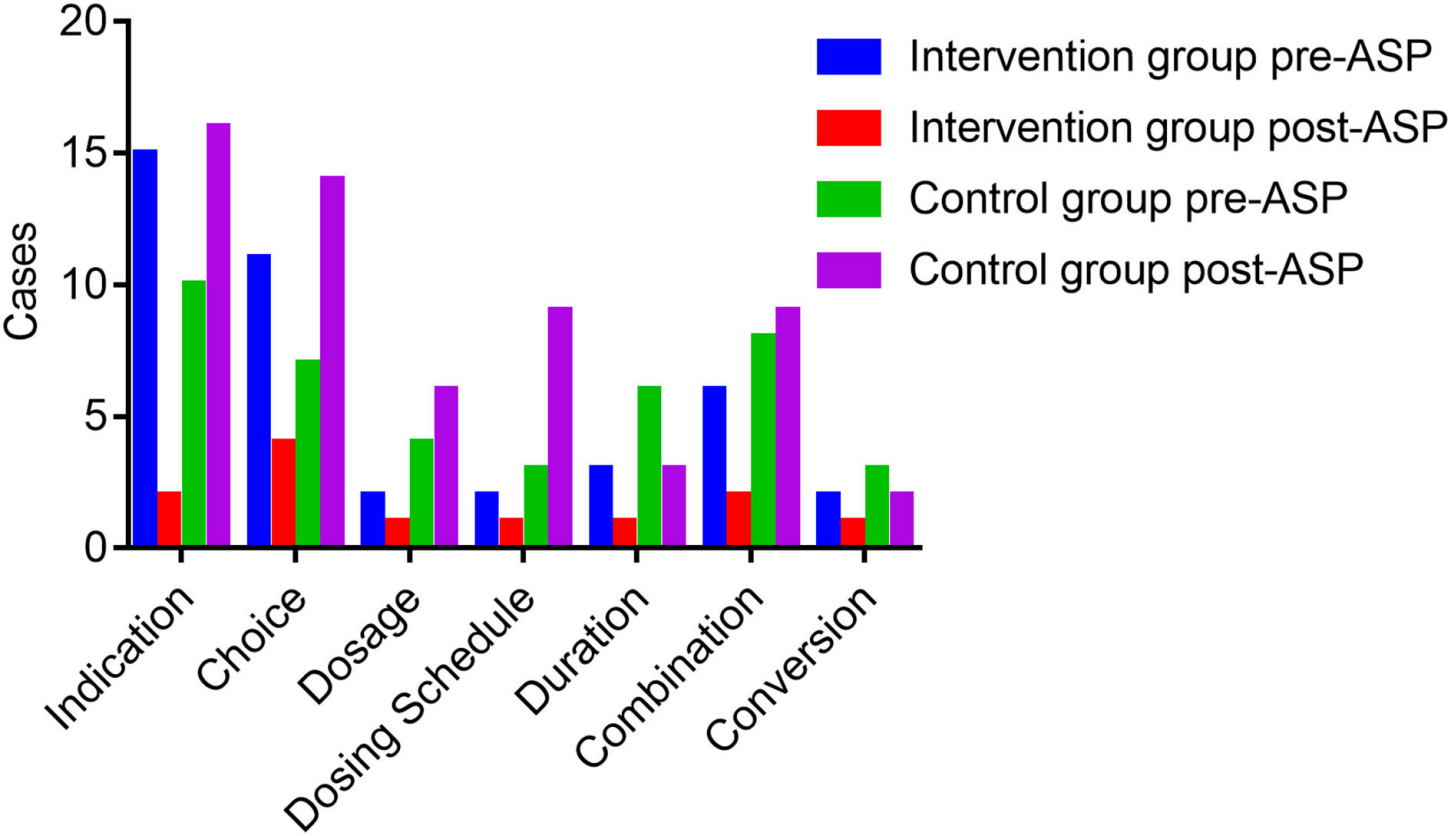
Inappropriate use of antibiotics before and after the ASPs implementation in the respiratory ward. ASPs, antimicrobial stewardship programs.

**Figure 3:**
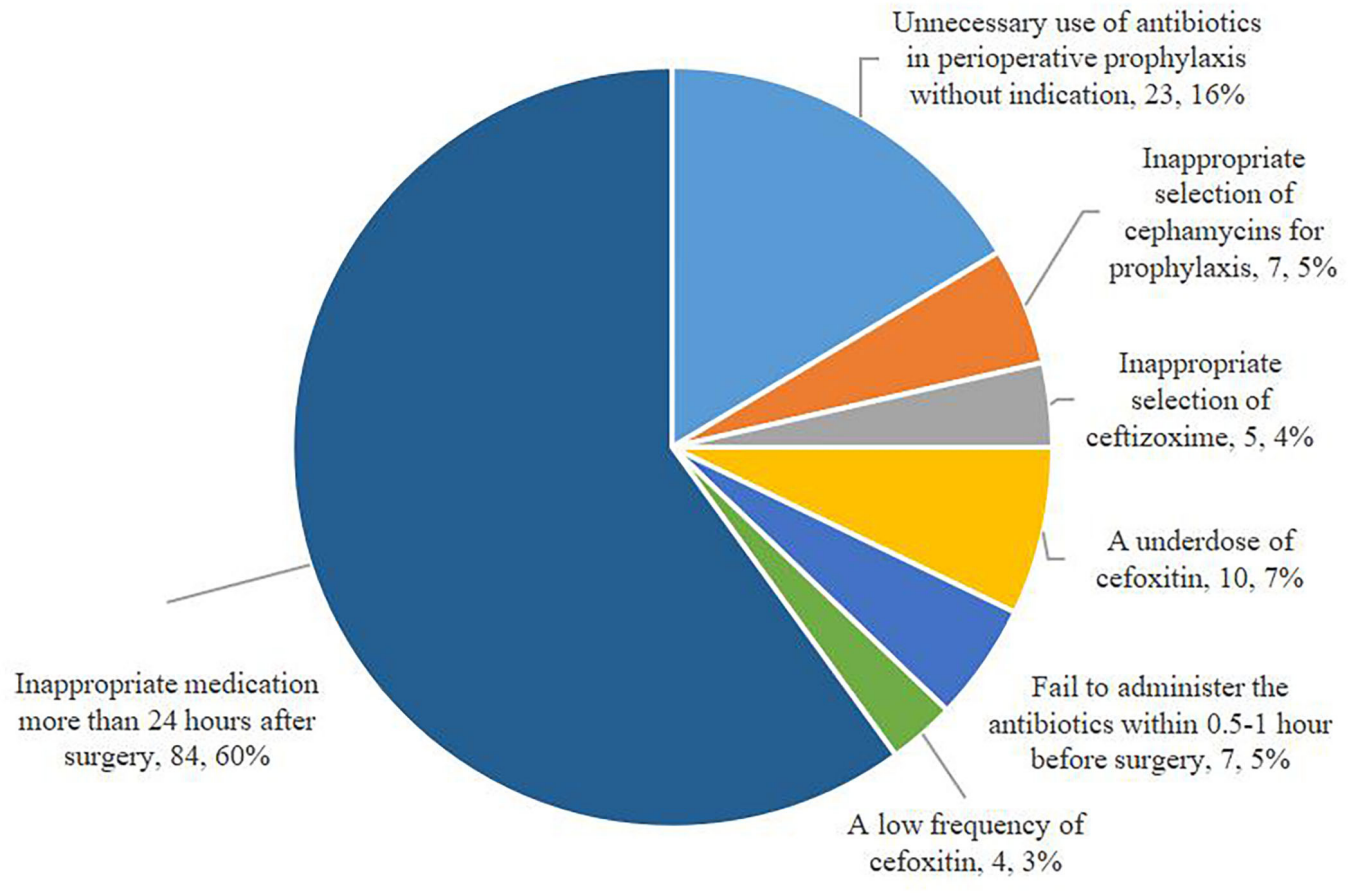
Inappropriate perioperative antimicrobial prophylaxis in the hepatobiliary surgery ward.

**Figure 4:**
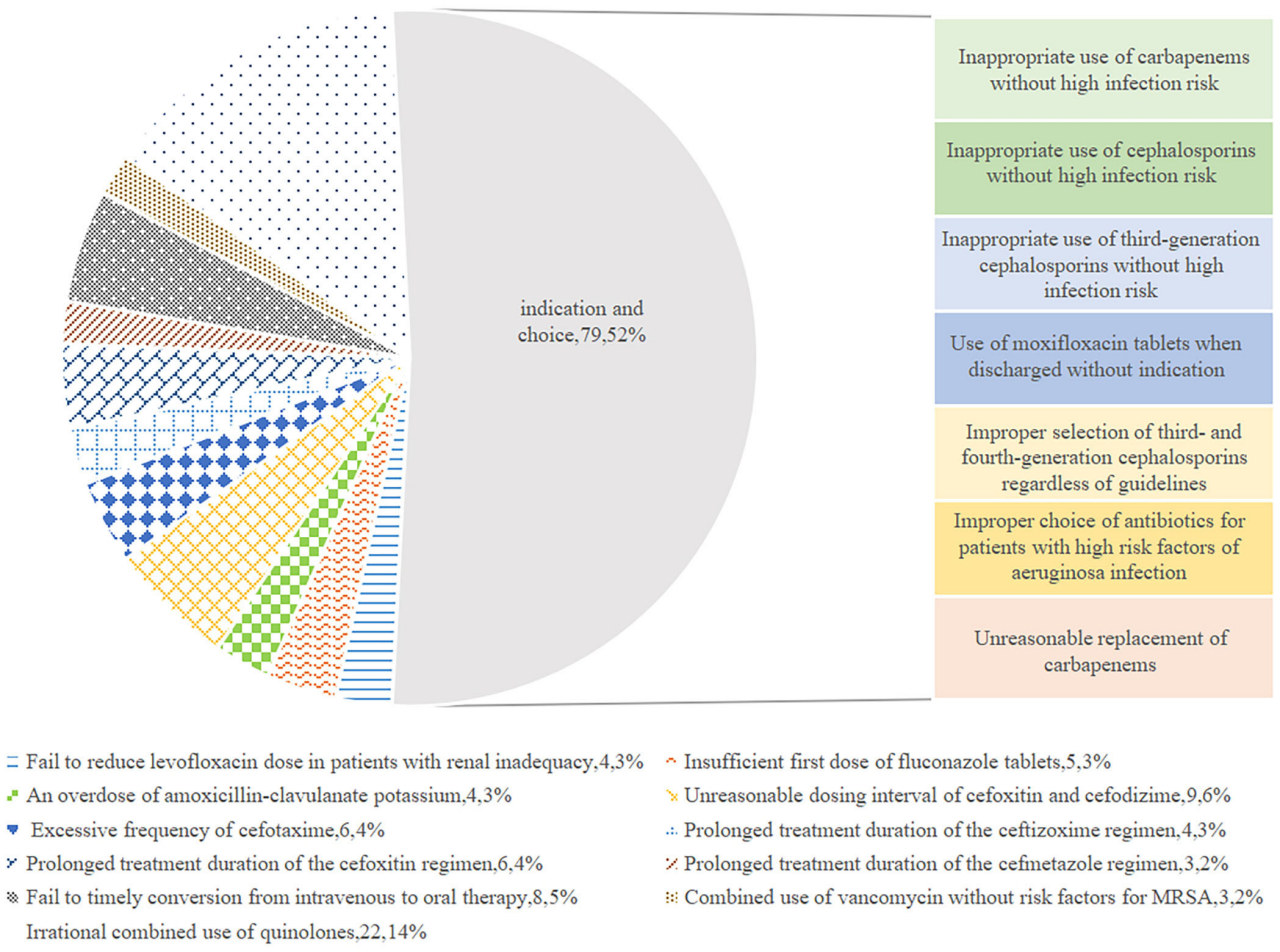
Inappropriate therapeutic use of antibiotics in the respiratory ward.

Figure 1 exhibits the inappropriate use of antibiotics in the hepatobiliary surgery ward. Most cases were identified as inappropriate duration, followed by irrational indication. During the pre-ASP period, 12 cases in the intervention group and 18 cases in the control group were identified as an unnecessary duration for preventive medication, and after the ASPs, the cases of inappropriate duration decreased in the intervention group, but increased in the control group. In addition, the cases with inappropriate indication, choice, and dosing schedule in the intervention group were fewer than the cases in the control group after ASP implementation. However, regarding the dosage item, the cases in the intervention group were more than the cases in the other group. As shown in Figure 2, most cases were identified as inappropriate indications of the use of antibiotics, followed by irrational choice and unnecessary combination use of antibiotics. The cases with irrational use of antibiotics in the intervention group declined dramatically after the ASPs implementation as expected, and those with inappropriate indication decreased from 15 to 2 cases. Except for the conversion item, the cases in the intervention group decreased while having an increasing trend in the control group before and after the intervention. The main inappropriate use of antibiotics in two wards is shown in Figures 3, 4, respectively.

## Discussion

The previous evidence demonstrated that the temporary absence of pharmacists in the program has resulted in a prolonged duration of therapy, more than the 3-fold number of cases of CDI, and a 27–39% increase in inappropriate use of antimicrobial agents (14). Besides, a study showed that the appropriateness of approvals for restrictive antibiotics performed by infection pharmacists was better than that performed by infection physicians (15). The ASPs in this study were supported by hospital leadership and heavily focused on reviewing prescriptions and restricting antibiotics to affect the antimicrobial prescribing behavior of physicians. Slightly different from other studies, the reward and punishment mechanism which is capable of catalyzing improvement in efforts on rational use of antibiotics (16) was established in this study. After a 10-month ASP, the observed results of reduction in antibiotic consumption and costs and improvement in appropriate use provide additional evidence to support the positive effects of pharmacist-led ASPs in China.

In general, consistent with other findings (17), our result demonstrated the ASPs intervention resulted in a reduction in DDDs per patient and DDDs per patient per day in the respiratory ward and a decrease in DDDs per patient in the hepatobiliary surgery ward, indicating that the ASPs intervention was associated with a reduction in antibiotic consumption. Specifically, compared with the hepatobiliary surgery ward, a larger decrease in antibiotics consumption would be observed in the respiratory ward as the patients admitted to the ward were more likely to have a high rate of nosocomial infection and drug-resistant infection. Regarding antimicrobial costs, we observed a decrease in hospitalization costs in the surgery ward and a 22.26% reduction in antibiotics costs in the respiratory ward. Several reasons can account for the finding: First, the decrease in costs may be associated with a reduction in antimicrobial consumption. Second, as an important part of the antimicrobial stewardship team, microbiologists are responsible for cumulative antimicrobial susceptibility tests for the accuracy of antibiotics, and the shortened duration of empirical treatment due to clear microbiological results was a driver for cost saving. Third, the indicator of the average LOS in this study decreased after the ASPs intervention in the hepatobiliary surgery ward, which was similar to another study in the gastroenterology ward (18). Besides, LOS was a vital influencing factor for hospitalization costs. Meanwhile, the result showed a 4.74 and a 19.47% decline in daily costs after the ASPs implementation in the intervention group, whereas a non-significant decrease was observed through DID analyses in two wards, which suggested the average price of antibiotics used during the study period had not decreased as expected. This may be associated with a common misconception among patients that expensive drugs are more effective than cheap ones (19); a few physicians even were asked to prescribe expensive antibiotics under the pressure of patients.

One beneficial result was that the inappropriate combinations with β-lactams and fluoroquinolones improved in the intervention group after ASP implementation. In common practice, levofloxacin is often combined with cephalosporins or macrolides to treat common respiratory diseases such as chronic bronchitis. But for common infections, the combinations were less effective and an overdose of levofloxacin in the respiratory ward had the potential to result in bacterial resistance. Thus, it is prescribed that levofloxacin should be used only for unexplained infections, and severe infections cannot be improved by single medication, mixed infections, or MDROS infections (20), which contributed to the decrease in cases identified as improper combinations after the ASP intervention. Nevertheless, the high dosage of levofloxacin cannot be reduced for the patients with renal inadequacy in our study.

The other beneficial result demonstrated the reduced consumption and increased rationality of utilization of cephalosporins after the ASP intervention, although the cephalosporins are widely used in clinical departments as a result of the considerable antibacterial activity of them against gram-negative and gram-positive organisms and are consumed in great quantities before the implementation of ASPs. In consistent with the result of a previous study (21), the consumption of cephalosporin compound medicines such as cefoperazone and sulbactam has been reduced through academic education lectures and training courses. Meanwhile, a decrease in the consumption of cephalosporins was observed after the intervention. As the causative agent of surgical infection, the widespread resistance of Escherichia coli to ceftriaxone was revealed in the hepatobiliary surgery ward, which accounted for the reduction in ceftriaxone prescriptions.

Another finding showed that timely conversion from intravenous to oral therapy improved in the intervention group (22). The obstacle to conversion therapy is the deep-rooted misconception that intravenous infusion is more effective than oral drugs (23), which is caused by low Chinese health literacy. In this study, pharmacists addressed that if the oral antibiotics showed excellent oral bioavailability and strong tissue penetration, the efficacy would be comparable to those attained by intravenous infusion.

One interesting finding showed that there was an increase in DDDs per patient and a decrease in antibiotic costs in the hepatobiliary surgery ward after the intervention. On the one hand, the increase in DDDs per patient might be caused by prolonged average LOS. The longer hospital days, which might contribute to more antibiotics, were prescribed. On the other hand, due to the implementation of ASP, low-grade or high-grade physicians were authorized to prescribe different classes of antibiotics, and first-line antibiotics should be recommended as the priority prescription. Therefore, physicians usually prescribe cheaper first-line antibiotics than expensive second-line or third-line antibiotics during the ASPs intervention period.

However, there still existed inappropriate use of broad-spectrum antibiotics, which suggested that the improvement in prescribing behavior of physicians was a tough assignment in antimicrobial management. Theoretically, the antibiotics' effectiveness and resistance development are supposed to be taken into consideration by guidelines when giving medication to patients. Nevertheless, physicians have a different thinking process that they would attach more importance to the effects of antibiotics than the spread of resistance in empirical use (24), which results in the overuse of broad-spectrum antibiotics such as meropenem and biapenem. Meanwhile, the concerns of physicians about the failure of the treatment can also lead to the excessive use of broad-spectrum antibiotics for surgical prophylaxis and frequent switching and combination of antibiotics (18). It is common for some patients without proper indications to require prescriptions for antibiotics (25). For example, although no clinical indication for parenteral therapy, most physicians still would prescribe injectable antibiotics under the pressure from patients and their parents (26).

Although the pharmacists performed the leading role in ASPs, the participation of pharmacists was limited to monitoring antimicrobial use, communicating with physicians and patients on therapy, providing feedback with clear evidence to prescribers, developing medication guidelines, and educating medical staff during the study period, which indicated that the pharmacists played fewer clinical roles than they might otherwise. This may be associated with the limited number of trained pharmacists performing in clinical departments and overburdened pharmacists having difficulty taking various crucial clinical roles (27). Therefore, sustainable formal education and clinical practice ought to be provided for pharmacists to improve their experience in optimizing antimicrobial use and therapy. Additionally, education with different models of reflection on practice and group learning and discussion are introduced to change prescribing behaviors of physicians (28). Last but not least, to maintain the effectiveness of ASPs, more funding and staff support should be provided to overcome the barriers (29).

Our study had several strengths. This study avoided the selection bias as the patients in the control group have similar characteristics to the intervention group through the PSM method, and a DID model has the potential to control the confounding influences of dependent variables and solve the endogeneity problem. However, several limitations should be acknowledged in this study. First, although the PSM was used to balance confounding variables, some unmeasured variables might cause different trends between the intervention and control groups. Second, the respiratory wards and hepatobiliary surgery wards were served by two independent pharmacists and the ASPs strategies were performed in the intervention group in each ward; however, the potential of the influence on the education and training of physicians in the control group was unavoidable. Third, due to a series of strategies included in ASPs, the impact affected by which strategy cannot be accurately assessed. Finally, the impact of clinical outcomes such as mortality, reinfection rate, and resistance rate cannot be estimated as the relevant data were not collected, and we were unable to assess the long-term effects of the ASPs due to the short study period.

## Conclusion

This study provides a DID analysis of the impacts of the ASPs involving a pharmacist and reveals that the ASPs are effective in reducing the length of hospital stay, decreasing antibiotics consumption and costs, and improving the appropriateness of antimicrobial use such as decreasing irrational use of cephalosporins, reducing combinations, and improving timely conversion. Nevertheless, it has failed to reduce the daily costs of patients and decrease the improper use of broad-spectrum antibiotics. The government must provide sustainable formal education and clinical practice for pharmacists and more funding and staff support to optimize the structure and promote the construction of ASPs.

## Data availability statement

The original contributions presented in the study are included in the article/supplementary material, further inquiries can be directed to the corresponding authors.

## Author contributions

YW, CL, SL, XLL, and XL conceptualized and designed the whole study. YW, CL, SL, XLL, and CZ were responsible for collecting and analyzing the data. YW drafted the initial manuscript. XL took the responsibility for editing. All authors contributed to the critical revision of the manuscript and approved the final version.

## Funding

This work was supported by the National Natural Science Foundation of China (71673147 and 72074123) and the China Medical Board (Grant no. 17-277).

## Conflict of interest

The authors declare that the research was conducted in the absence of any commercial or financial relationships that could be construed as a potential conflict of interest.

## Publisher's note

All claims expressed in this article are solely those of the authors and do not necessarily represent those of their affiliated organizations, or those of the publisher, the editors and the reviewers. Any product that may be evaluated in this article, or claim that may be made by its manufacturer, is not guaranteed or endorsed by the publisher.

## Author disclaimer

The contents are solely the responsibility of the authors and do not reflect the views of the funding bodies or any organization.
